# Laboratory Automated Interrogation of Data: an interactive web application for visualization of multilevel data from biological experiments

**DOI:** 10.1093/braincomms/fcae074

**Published:** 2024-02-29

**Authors:** Owen R Dando, Zrinko Kozic, Sam A Booker, Giles E Hardingham, Peter C Kind

**Affiliations:** Simons Initiative for the Developing Brain, University of Edinburgh, Edinburgh, EH8 9XD, UK; Patrick Wild Centre, University of Edinburgh, Edinburgh, EH8 9XD, UK; UK Dementia Research Institute, University of Edinburgh, Edinburgh, EH16 4SB, UK; Centre for Discovery Brain Sciences, University of Edinburgh, Edinburgh, EH8 9XD, UK; Simons Initiative for the Developing Brain, University of Edinburgh, Edinburgh, EH8 9XD, UK; Centre for Discovery Brain Sciences, University of Edinburgh, Edinburgh, EH8 9XD, UK; Simons Initiative for the Developing Brain, University of Edinburgh, Edinburgh, EH8 9XD, UK; Patrick Wild Centre, University of Edinburgh, Edinburgh, EH8 9XD, UK; Centre for Discovery Brain Sciences, University of Edinburgh, Edinburgh, EH8 9XD, UK; Simons Initiative for the Developing Brain, University of Edinburgh, Edinburgh, EH8 9XD, UK; Patrick Wild Centre, University of Edinburgh, Edinburgh, EH8 9XD, UK; UK Dementia Research Institute, University of Edinburgh, Edinburgh, EH16 4SB, UK; Centre for Discovery Brain Sciences, University of Edinburgh, Edinburgh, EH8 9XD, UK; Simons Initiative for the Developing Brain, University of Edinburgh, Edinburgh, EH8 9XD, UK; Patrick Wild Centre, University of Edinburgh, Edinburgh, EH8 9XD, UK; Centre for Discovery Brain Sciences, University of Edinburgh, Edinburgh, EH8 9XD, UK

**Keywords:** data, visualization, automatization, statistics, R Shiny

## Abstract

A key step in understanding the results of biological experiments is visualization of the data. Many laboratory experiments contain a range of measurements that exist within a hierarchy of interdependence. An automated and facile way to visualize and interrogate such multilevel data, across many experimental variables, would (i) lead to improved understanding of the results, (ii) help to avoid misleading interpretation of statistics and (iii) easily identify outliers and sources of batch and confounding effects. While many excellent graphing solutions already exist, they are often geared towards the production of publication-ready plots and the analysis of a single variable at a time, require programming expertise or are unnecessarily complex for the task at hand. Here, we present Laboratory Automated Interrogation of Data (LAB-AID), an interactive tool specifically designed to automatically visualize and query hierarchical data resulting from biological experiments.

## Teaser

Dando *et al.* present Laboratory Automated Interrogation of Data (LAB-AID), an interactive software tool to visualize and query hierarchical data resulting from biological experiments. LAB-AID helps biologists to understand results, identify outliers and sources of batch and confounding effects and avoid common statistical errors such as pseudoreplication.

## Introduction

There is a great need for biologists to visualize and interrogate data from their laboratory experiments, for both exploration and communication of results, and to avoid erroneous conclusions being drawn from automated analyses.^1-4^ This is particularly true when measurements exist within a hierarchy of interdependence and when multiple sources of batch and confounding effects may influence experimental results.

For example, in a common type of experiment in the translational neuroscience field, such as an investigation into the electrophysiological or histological properties of neurons in the brain of a transgenic rodent model of a neurodevelopmental or neurodegenerative condition, diverse measurements are made on individual neurons whose behaviour is not independent of each other (e.g. these neurons were subject to the same developmental programme that gave rise to a functioning brain).^5^ From a single animal, multiple neurons may be probed within an individual brain slice or multiple slices. Furthermore, multiple animals will be tested which may or may not be litter mates or have shared cages; animals from the same litter or cage will not be independent, because they have shared the same parents and prenatal and/or postnatal environment.^6^ Random effects (such as slice, animal, litter or cage) at any level within this hierarchy can consistently impact groups of measurements, possibly leading to misinterpretation of results if ignored.^7^

Moreover, data may be gathered by different investigators on several dates, using diverse experimental equipment, resulting in potential batch effects that may further complicate interpretation. These random and batch effects can be difficult to disentangle from the factors being investigated. In addition, it is often important to understand the relationship or correlation between different measurements made on the same experimental unit (for example, multiple different electrophysiological measurements made on each neuron or multiple behavioural measurements made on each animal) or to identify if a neuron (or slice, animal, litter or cage) is an outlier with respect to one or to many experimental variables.

Thus, it is important to thoroughly visualize and understand such data before proceeding to interpret numerical analyses or statistical tests of effects; otherwise, misleading, inappropriate or even spurious conclusions may be drawn. The proper visualization of the data can also help researchers guard against common statistical errors (e.g. pseudoreplication^5^). In addition, the identification of outliers at all levels within the hierarchy of measurements may help to determine cases of erroneous measurement or recording of data.

While numerical and statistical computing environments such as R and MATLAB provide a rich and powerful framework within which experimental data can be examined and plotted, their use is not necessarily straightforward for those without experience or training in programming. Dedicated scientific graphing software tools such as GraphPad Prism^8^ or JASP^9^ provide graphical user interfaces which may be more approachable for the laboratory scientist; a number of online tools have recently been developed to automatically plot experimental data provided in the form of a spreadsheet or table,^10-17^ and more general online plotting tools are available (such as Plotly Chart Studio^18^ and RAWGraphs).^19^ However, such software may be more oriented towards examining a single experimental variable at a time, to producing publication-ready plots or a wide range of detailed statistical analyses, towards providing a wide range of general features and plot types at the price of complexity and time spent learning their use or require setup and configuration effort for each plot produced.

Hence, we believe it would be of considerable use to laboratory scientists to have a tool which could automatically import and plot, without the need for further setup or configuration, such multilevel, multivariable data from biological experiments, by which they could interactively examine distributions, correlations between variables, outliers and batch effects, at all levels within the hierarchy of measurements, and thus improve the rigour and reproducibility of their conclusions. Here we present Laboratory Automated Interrogation of Data (LAB-AID), a tool specifically designed to visualize and interrogate multilevel, multivariable data resulting from biological experiments.

## Materials and methods

### Example data

As a demonstration of LAB-AID, we use experimental data in which the morphology, function and integration of dendritic spines was studied in layer 4 spiny stellate cells from the somatosensory cortex of a mouse model of a monogenic neurodevelopmental disorder and compared with the behaviour of neurons of wildtype (control) mice.^20^ In the particular data used, intrinsic electrophysiological properties were measured on neurons from control and knockout mice; multiple cells were tested per brain slice and multiple brain slices per experimental animal.

### Technology

LAB-AID is implemented using Shiny, a package for the R programming language which allows interactive web applications to be built. Plots are produced with the ggplot2, ComplexHeatmap, and patchwork R packages, while interactive graph functionality is provided by the plotly package. Statistical analyses are performed with the car and lme4 packages, data and configuration are input and output with the jsonline, readxl and WriteXLS packages, and the user interface is supported with the DT, shinycssloaders, shinyjs and shinyWidgets packages. Also used internally for data manipulation are the magrittr and reshape2 packages.

### Input data format

Individual data sets for LAB-AID should take the form of an Excel spreadsheet (both ‘.xls’ and ‘.xlsx’ files are supported) or a comma-separated values (CSV) file. Data should be in wide format, in which each row comprises metadata and measurements of experimental variables on a single experimental unit ­­(for example, a cell or an animal—however, all rows in a single data set should refer to the same type of experimental unit).

Columns containing metadata should appear first. In the parlance of mixed modelling, these will include both fixed and random effects; for example, our example data set contains a column indicating the genotype of the animal from which each measured neuron was derived (a fixed effect) and also columns indicating the brain slice and animal ID for each cell (random effects). Entries in metadata columns must be provided for every experimental unit (that is, there should be no empty cells in the metadata columns).

Data columns should follow the metadata columns. Usually, multiple data columns will be present, representing measurements of multiple variables made on the same experimental units; for example, our example data set contains columns for resting membrane potential, membrane time constant, input resistance and a number of other electrophysiological variables that were measured for each neuron.

Note that not all measurements need to be recorded for every experimental unit (that is, the data columns may contain empty cells). For plots made using a single variable, an empty cell for a particular experimental unit means that unit will not contribute a value to the plot. For correlation heatmaps, an empty cell for a particular variable means that experimental unit will not contribute to pairwise correlations calculated between that variable and any other (though the experimental unit will still contribute towards correlations calculated between pairs of variables for which both measurement cells, respectively, do have values).

It is important to note that LAB-AID is wholly agnostic to the nature of both metadata and measurement columns and hence is equally applicable to data produced by different types of experiment (for example, electrophysiological, behavioural or molecular).

### Installation and configuration

#### RShiny application installation

LAB-AID is implemented using Shiny and thus can be installed or deployed in the same way as any other Shiny application. For example, the code can be run on the user’s own machine using RStudio, deployed to a web server using Shiny Server or, if local server resources are not available, deployed to the cloud using shinyapps.io (https://www.shinyapps.io)—in the latter two cases, deployment to a webserver allows multiple users to upload, examine and interrogate the same data sets simultaneously. We have also provided a Docker image hosted on Docker Hub (https://hub.docker.com) that contains a prebuilt web server running the LAB-AID application. Detailed instructions on how to run LAB-AID via any of these methods are provided on our GitHub repository (https://github.com/sidbdri/LAB-AID).

#### JavaScript Object Notation configuration

LAB-AID configuration is stored in a simple JSON (JavaScript Object Notation, https://json.org) file residing in the same directory as the application code, which describes the location and structure of the input data. The configuration file describes a number of data sets, each of which corresponds to a particular Excel spreadsheet or CSV file. For each data set, the following must be defined:

‘Name’: A name for the data set, which will appear in the LAB-AID user interface.

‘Path’: Path to the Excel or CSV file containing data for this set, relative to the location of the LAB-AID code directory.

‘n.Factors’: The number of metadata columns in this data set.

‘Description’ (optional): A description of the particular data set which will appear on the ‘About’ tab.

Note that while all data sets defined in the configuration file will be available for exploration in the user interface, data sets do not need to contain the same metadata columns, nor do they need contain measurements on the same experimental units (or type of unit).

In addition, the main application title and description are also configured in the appropriate JSON fields:

‘Title’: An overall title for the application which will appear at the left of the main tab bar.

‘About’: An overall description of the data sets presented which will appear on the ‘About’ tab.

The ‘Configuration’ tab within the application provides an interface to add and remove data sets and to modify the application title and description. Thus, there is no need to edit the configuration file manually; this can, however, be done if desired.

## Results

**Figure 1:**
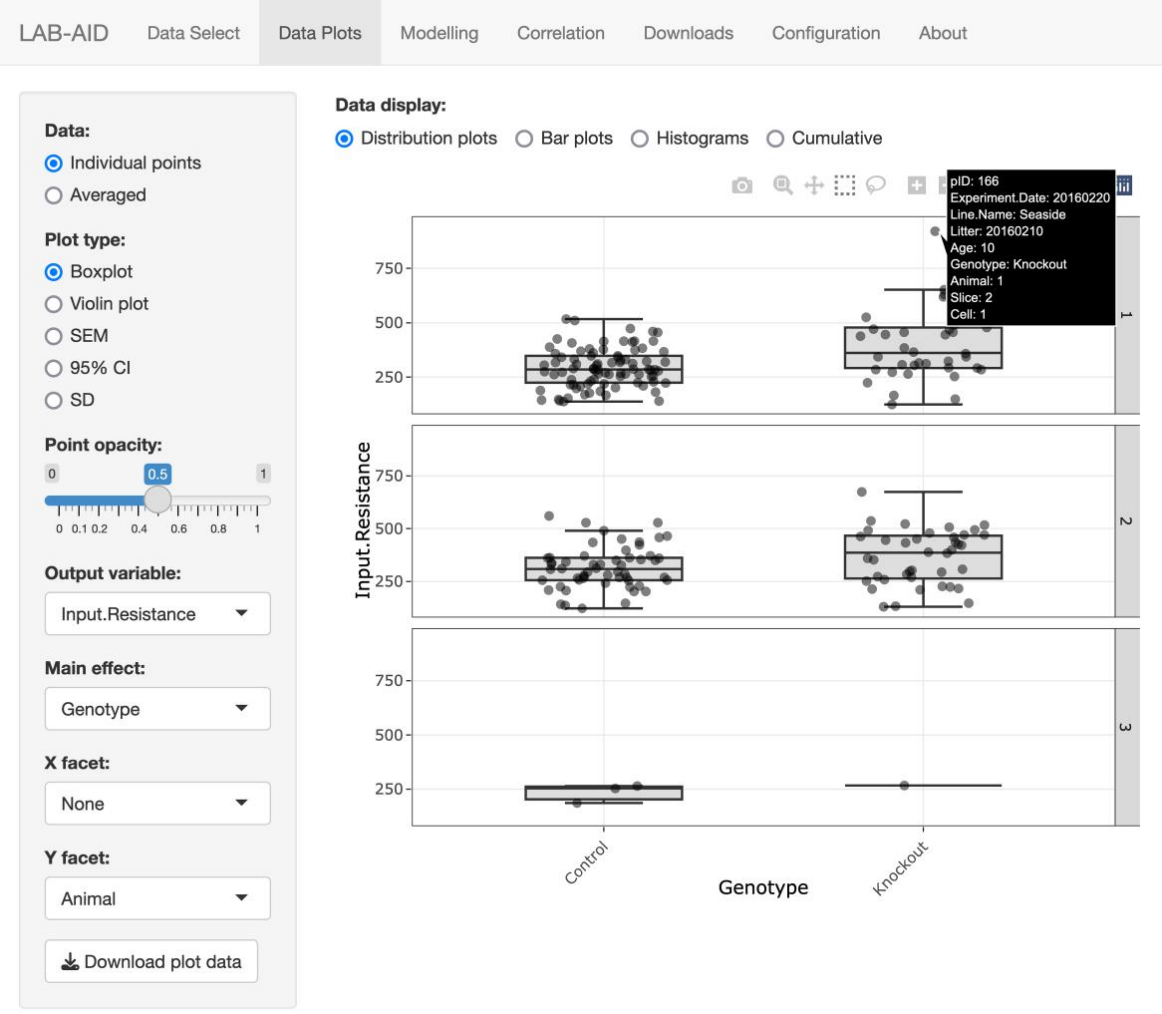
**The LAB-AID interface is split into seven major sections**. The Data Select, Data Plots, Modelling, Correlation, Downloads, Configuration and About sections are accessible from the main tab bar. Here, the ‘Data Plots’ tab is selected.

The LAB-AID user interface is split into seven major sections (see Fig. 1), available from the main tab bar, with different functions:

1. ‘Data Select’: Choose the data set, among those which are loaded, to be examined. Optionally select, depending on their metadata, which data points within the data set will be plotted and highlighted. Preview data selections and highlighting.
2. ‘Data Plots’: View automatically generated box or violin plots, bar plots, histograms or density plots or cumulative frequency distributions of chosen data, comparing between and across levels of fixed and random effects.
3. ‘Modelling’: Perform simple linear (mixed) model fitting and test the significance of fixed effects.
4. ‘Correlation’: View correlations between experimental variables, calculated across experimental units, and comparisons of correlations between different levels of particular effects.
5. ‘Downloads’: Export subsets of the input data for download.
6. ‘Configuration’: Configure the data sets available for investigation and modify the application title and description.
7. ‘About’: Read information describing the presented data sets.

### Data selection and highlighting

Data sets for LAB-AID take the form of an Excel spreadsheet or CSV file, in which each row comprises metadata and measurements of experimental variables on a single experimental unit (for example, a cell or animal); multiple such data sets may be loaded into the application at the same time. A drop-down selection box at the top of the ‘Data Select’ tab allows the user to choose the data set (of those currently loaded) to be examined. Below this, three further tabs give access to data selection, highlighting and preview functionality.

Next to the ‘Dataset’ selection box is a multiple selection drop-down box, ‘Averaging factors’, which allows the user to select metadata factors over which the measured variables can be averaged. A common use case in neuroscience experiments would be to allow measurements to be shown averaged for individual animals (i.e. animal means), but it also possible to average over different experimental conditions as desired, such as experimental dates or animal models. To achieve averaging over animals in the example data set, all factors except ‘slice’ and ‘cell’ should be selected. Whether individual or average measurements will be plotted can be selected in the sidebar menu on the ‘Data Plots’ tab (see below).

Note that the ‘Averaging factors’ box, along with all other user interface elements allowing the choice of experimental variables or metadata factors, is automatically populated from the data set being examined, without requiring any further configuration from the user.

#### Selecting data to be plotted

The ‘Selection’ subtab allows the user to choose which data are to be included in graphs. A drop-down selection box is automatically presented for each column of metadata in the current data set. These allow subsets of the data to be included in or excluded from plots, depending on the metadata characteristics of each experimental unit (for example, one might choose to exclude all cells derived from a particular animal). Individual levels of each metadata factor can be selected or deselected individually; in addition, ‘Select All’ and ‘Deselect All’ buttons allow for bulk selection and deselection. By default, all data in the data set are selected to be shown in plots.

#### Highlighting data subsets

The ‘Highlights’ subtab allows the user to choose which data, among that included in the graphs, are to be further highlighted in colour. Its user interface is analogous to that of the ‘Selection’ tab, with a drop-down selection box automatically presented for each column of metadata in the current data set; data points can then be highlighted according to the metadata characteristics of each experimental unit (for example, one might highlight all cells derived from a particular slice). By default, no data are selected to be highlighted.

#### Previewing data plots

The ‘Preview’ subtab presents a simple plot of the distribution of data for each experimental variable within the current data set. Individual data points are shown, and overall distributions can be represented as either box or violin plots. Data are graphed according to the different levels of a single factor, chosen from the metadata columns in the input data. Preview plots respect the selection and highlighting choices made in the ‘Selection’ and ‘Highlights’ tabs.

Hovering over any data point in the ‘Preview’ tab (or in the main ‘Data Plots’ tab) will display a tooltip showing metadata attributes for that data point. In addition to all the metadata derived from the input Excel or CSV file, a point ID (pID) attribute is shown, which corresponds to the relevant row in the input file, thus allowing easy location of points of interest within the input data file. The pID attribute can also be used to easily highlight particular points or exclude them from plots.

### Data plots

The behaviour of a particular experimental variable can be examined in detail in the top-level ‘Data Plots’ tab. The main area of the tab shows either (i) data distribution plots, (ii) bar plots, (iii) histogram or density plots or (iv) cumulative frequency plots, for a particular experimental variable of interest; choice between these four modes is made via the ‘Data display’ radio buttons. At the left of the ‘Data Plots’ tab, a sidebar provides further control over the plots shown in the main area. Some sidebar elements are common to all plot types, whereas others change depending on the plot type.

Among the common controls, the ‘Data’ radio buttons determine whether the data plotted is for individual experimental units (i.e. for each row in the input data file) or if it should be averaged over the levels of metadata factors selected in the ‘Averaging factors’ selection box under the ‘Data Select’ tab. The last four selection boxes control which variable will be plotted (‘Output variable’) and how data should be subdivided for comparison. The ‘Main effect’ selection box determines the metadata factor of primary interest; how this is represented depends on the type of plot being shown and will be further described below. The ‘X facet’ and ‘Y facet’ selection boxes allow for the further subdivision of the data into separate columns and rows of graphs, respectively, according to the levels of two additional metadata factors.

While extensive data download functionality is provided by the top-level ‘Downloads’ tab, it may be useful to extract solely the data that contributes to a particular plot that is being examined (for example, to prepare that exact data for a publication-ready plot, in a tool such as GraphPad Prism^8^). An Excel spreadsheet containing the data for the current plot can be obtained by clicking the ‘Download plot data’ button.

#### Distributions

**Figure 2:**
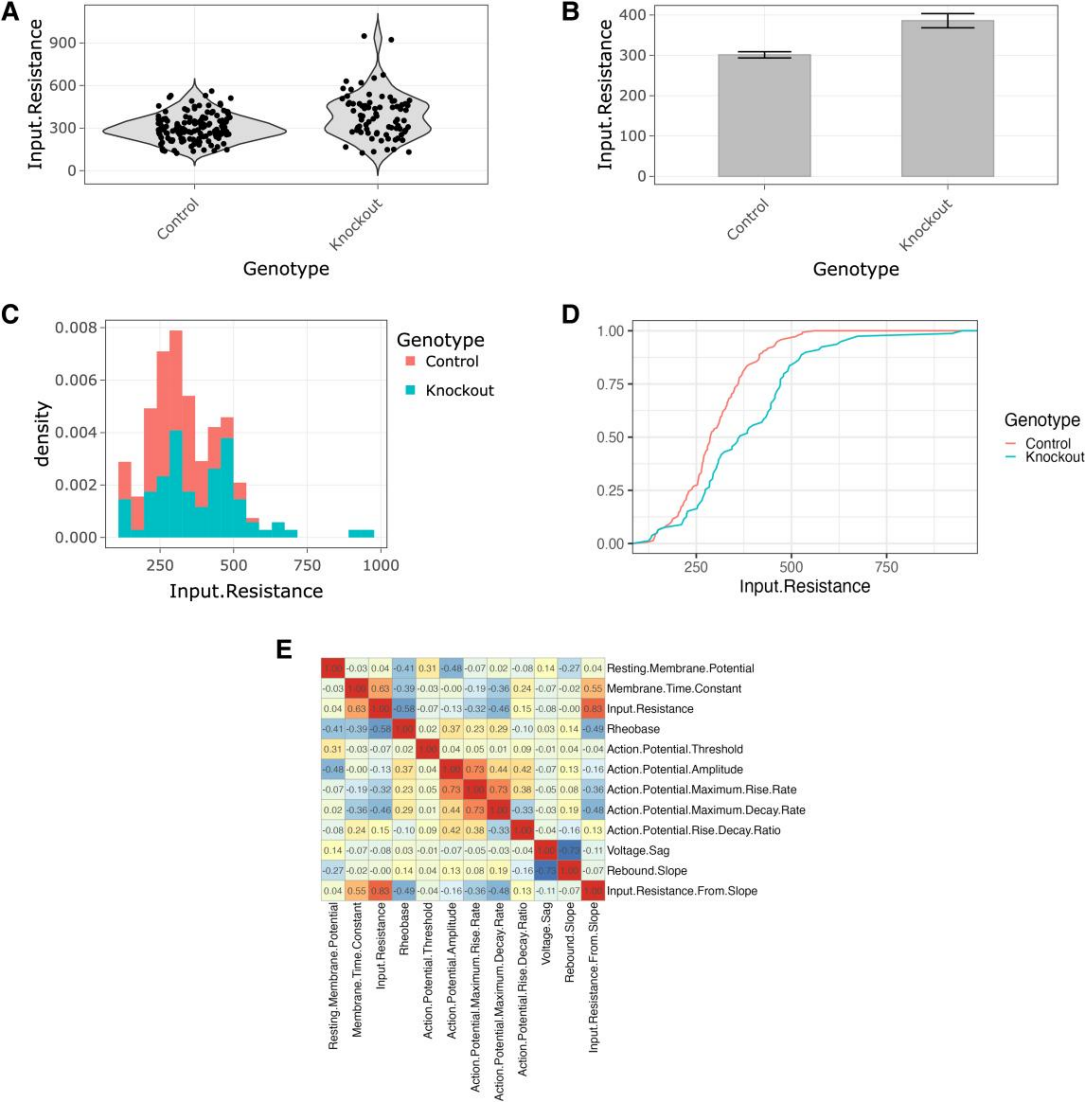
**The behaviour of experimental variables can be examined in detail by a range of automatically generated plots**. Shown are examples of (**A**) violin plots, (**B**) bar plots, (**C**) histograms, (**D**) cumulative frequency distributions and (**E**) correlation heatmaps. All plots are generated automatically from the loaded data set and are immediately available through the LAB-AID interface, with no further setup or configuration required by the user.

If the ‘Distribution plots’ radio button is selected, then individual data points (or averages) for the chosen experimental variable are plotted, along with overall distributions (see Fig. 2A); data selection and highlighting choices made in the ‘Data Select’ tab are respected. In each graph displayed, data are separated on the *x*-axis into separate levels of the main effect chosen in the left sidebar, and the overall distribution shape is shown for each. If no selections are made in the ‘X facet’ and ‘Y facet’ selection boxes, then a single graph is shown containing all data. In the sidebar, radio buttons specific to this plot type control how overall distributions will be represented. This can be in form of a box or violin plot or a scatter plot including error bars corresponding to standard error of the mean (SEM), 95% confidence interval (95% CI) or standard deviation (SD).

#### Bar plots

Selecting the ‘Bar plots’ radio button displays summary statistics for the selected experimental variable in a form of a bar plot, where the height of the bar corresponds to the mean value of the variable (see Fig. 2B). By selecting the corresponding option in the sidebar menu, error bars can be set to display either the SEM, 95% CI or SD. Similarly to distribution plots, bars are initially split by the main effect and the plot can be faceted by additional variables. Bar plots can also be generated based on individual data points or averaged over factors selected in the ‘Averaging factors’ selection box.

#### Histograms

When the ‘Histograms’ radio button is selected, data for the chosen experimental variable are plotted as a histogram or density plot, as determined by the ‘Plot type’ radio buttons in the sidebar (see Fig. 2C). If data are plotted as a histogram, then the ‘Bins’ slide control in the sidebar determines the number of bins. Data are separated by colour of bars (histogram) or line (density plot) into different levels of the main effect chosen in the sidebar. If no selections are made in the ‘X facet’ and ‘Y facet’ selection boxes, then a single histogram or density plot is shown representing all data; otherwise, the data can be subdivided into multiple histograms or density plots according to the levels of one or two extra factors.

#### Cumulative frequency distributions

Finally, if the ‘Cumulative’ radio button is selected, data for the chosen experimental variable are plotted as a cumulative frequency distribution (see Fig. 2D). Data are separated by colour of line into different levels of the main effect chosen in the sidebar. As before, a single plot encompassing all data can be shown, or the data can be subdivided according to one or two additional factors. There are no plot type-specific controls in the sidebar for cumulative frequency distributions.

### Modelling

LAB-AID allows users to perform simple statistical testing on data sets by fitting linear (mixed) models. While this functionality should not replace detailed modelling and analysis, which requires statistical expertise and dedicated statistical software, it can allow users to quickly assess the likely magnitude of effect sizes and to investigate the possible influence of modelling the levels of the hierarchy of interdependence of their data on calculations of statistical significance.

In the top-level ‘Modelling’ tab, linear model and linear mixed model fitting are performed, respectively, using the ‘lm’ and ‘lmer’ functions from the R package lme4. Which variable is to be modelled can be chosen from the ‘Select variable’ drop-down menu. Selecting the ‘Log-transformed’ checkbox will log-transform this variable (and if the data contains any non-positive values, a warning will be displayed to indicate that the log-transformation cannot be applied). Below these data selection and transformation options, a quantile–quantile (QQ) plot of the selected variable is displayed. The QQ plot can help to assess how well the raw or log-transformed data fits a normal distribution; data with error distributions other than the normal distribution may require fitting to a generalized linear or generalized linear mixed model, which is beyond the scope of LAB-AID’s statistical functionality.

In the right-hand column of the tab, users can select a single main factor or fixed effect to be tested for the selected variable. Below this, the ‘Select random factors’ box allows multiple random factors to be added to the model; in mixed models, these random factors can explain sources of variation which do not derive from the fixed effect. If no random factors are selected, the variable data will be fitted with a linear model using the ‘lm’ function. If one or more random factors are selected, the data will be fitted with a linear mixed model using the ‘lmer’ function. Note that if more than one random factor is selected, the factors will be nested in the order that they appear in the input CSV or Excel file.

At the bottom of the ‘Modelling’ tab, the output from the ‘lm’ or ‘lmer’ model fitting function is shown. Estimates of statistical significance of the fixed effect are provided either by the output of ‘lm’ or, in the case of mixed modelling, by the output of the ‘Anova’ function from the R package car.

### Correlations

The top-level ‘Correlations’ tab allows the investigation of associations between experimental variables (see Fig. 2E). Pearson correlations are calculated between each pair of variables, calculated over all experimental units by default (but respecting the data selections made on the ‘Data Select’ tab). Selecting an effect allows the user to subset the correlation calculation to those experimental units belonging to a particular level of a metadata factor (the effect and level are chosen from selection boxes in the sidebar at the left of the main plot). Correlations are drawn as a heat map, where colours run from blue (strong negative correlation) to red (strong positive correlation); the correlation value between each pair of variables is also shown.

If the ‘Pairwise’ check box is selected, then two heat maps are displayed, allowing the comparison of variable correlations calculated from different subsets of the data, defined by two levels of the chosen metadata factor. This allows changes in correlation between experimental conditions to be examined.

Finally, when the ‘Clustered’ check box is selected, then hierarchical clustering of experimental variables is performed, grouping together variables which show similar patterns of correlation against all the other variables. Otherwise, variables are displayed along the *x*- and *y*-axes of the heatmap in the order that they are defined in the input data file.

### Downloads

The top-level ‘Downloads’ tab allows subsets of the input data to be exported as a new Excel spreadsheet or CSV file. The two boxes at the top of the page determine what combination of metadata and experimental variable columns will be present in the exported file; the table at the bottom of the page previews the data columns that will be exported. Data are exported via the ‘Download.xlsx’ button as an Excel spreadsheet or ‘Download.csv’ button as an CSV file.

### Configuration

The top-level ‘Configuration’ tab allows the user to add and remove data sets from the application. To add a new data set, the user can upload a CSV file or an Excel spreadsheet containing data which adheres to the correct wide data format (see Materials and methods). The only further configuration required is a data set name, the number of metadata factors and optionally a data set description. The newly added data can then be immediately accessed on the ‘Data selection’ tab; all user interface elements will be automatically and appropriately populated, driven by the input data.

In the right column of the ‘Configuration’ tab, the ‘Update dataset’ interface allows users to upload an updated spreadsheet and select the name of the data set to be replaced from a drop-down menu. To remove a data set, the user can simply select the data set from the ‘Remove dataset’ drop-down menu and press the ‘Remove’ button.

At the bottom of the tab, there are options to change the application title, which is displayed at the left of the main tab bar, and the overall application description text, which appears on the top-level ‘About’ tab. Note that changes to text on the ‘About’ tab will only take effect the next time the application is launched.

### Use cases

We briefly highlight, in the context of the demonstration electrophysiological data presented here, a number of situations that LAB-AID makes simple to examine, with only a few mouse clicks:

- After noting a neuron that is an outlier with respect to one electrophysiological measurement, one can choose that data point—by its unique pID—to be highlighted and thus immediately see if the neuron is also an outlier with respect to other experimental variables.
- By highlighting data points originating from particular animals or litters, for example (or those data points which are commonly identified by any other metadata factor), one can examine whether that animal or litter appears to be driving one or more apparent phenotypes or if data originating that animal or litter are outliers with respect to one or a range of experimental variables.
- By excluding data points originating from particular animals or litters (or, again, points which are commonly identified by any other metadata factor), one can examine what effect this exclusion has on the means and variability of various experimental variables or on measures of statistical significance of metadata factors or whether correlations between electrophysiological measurements on neurons are robust to the removal of data originating from that animal or litter.

## Discussion

There is a great demand from biologists to be able to easily visualize, examine and interrogate the data from their laboratory experiments. Here, we have presented LAB-AID, a simple, yet powerful, tool specifically designed to automatically visualize hierarchical data from biological experiments, where a minimum of configuration is required before data interrogation can begin.

Within our own research groups, we use a wide range of graphing solutions. For example, our computational teams produce bespoke analyses using R and MATLAB, and many laboratory scientists use tools such as GraphPad Prism^8^ to produce publication-ready plots and perform statistical analyses. However, individual researchers also run LAB-AID on their own computers to quickly examine, interrogate and thus comprehend the data from the experiments they perform. We have also deployed instances of LAB-AID on a web server, accessible through the web browser by all researchers, onto which data sets from across the laboratory are loaded; these allow for individual or communal examination and interactive interrogation of data sets produced by our groups, for example, in lab meetings.

We also note that we have accrued further incidental benefit by adopting the standardized input data format required by LAB-AID for data produced within the laboratory, since this data can then also easily be read into programming environments such as R, for more bespoke analyses, without the need for tedious and time-consuming data cleaning and preparation steps.

## Data Availability

LAB-AID and the example data illustrated are available for download from GitHub under the permissive MIT Licence, at https://github.com/sidbdri/LAB-AID. Experienced R developers may thus freely extend or modify LAB-AID’s functionality according to their own needs.
